# Cytomegalovirus seronegativity rate in pregnant women and primary cytomegalovirus infection during pregnancy in rural Germany

**DOI:** 10.1186/s12884-023-05612-7

**Published:** 2023-04-28

**Authors:** Hannah Greye, Thomas Wex, Elina Taneva, Anke Redlich, Serban-Dan Costa, Anke Rissmann

**Affiliations:** 1 Malformation Monitoring Centre Saxony-Anhalt, Medical Faculty Otto-von-Guericke-University, Leipziger Straße 44, D-39120 Magdeburg, Germany; 2 Medical Laboratory for Clinical Chemistry, Microbiology, Infectious Diseases and Genetics “Prof. Schenk/Dr. Ansorge & Colleagues”, Schwiesaustraße 11, D-39124 Magdeburg, Germany; 3 Department of Obstetrics and Gynaecology, University Hospital Magdeburg, Gerhart-Hauptmann-Strasse 35, D-39108 Magdeburg, Germany

**Keywords:** Cytomegalovirus, CMV, CMV seroprevalence rate, Primary cytomegalovirus infection, Pregnancy, Seroconversion

## Abstract

**Background:**

Congenital cytomegalovirus (CMV) infection is the most common congenital infection worldwide and one of the leading causes of congenital hearing loss in newborns. The aim of this study was to determine the seroprevalence rate for cytomegalovirus in pregnant women and the rate of CMV serological testing utilised during pregnancy in a rural region in Germany.

**Methods:**

Retrospective data on the prevalence of CMV IgG and IgM antibodies were obtained from 3,800 women, identified in the study group of 19,511 pregnant women from outpatient settings whose samples were collected between 1 and 2014 and 30 April 2018. In addition, the serological CMV status in regards to various billing methods was further analyzed.

**Results:**

Serological CMV tests were performed in 3,800 (19.5%) out of 19,511 pregnant women. 2,081 (54.8%) of these women were CMV seronegative. Among those, seroconversion rate of 0.37–1.42% was identified. A proportion of 2,710 (14.7%) of all 18,460 women with statutory health insurance made use of the CMV testing as an individual health service.

**Conclusions:**

The low uptake of CMV serological testing in the study population covered indicates low risk awareness among pregnant women and their healthcare professionals. Presented seronegativity rates and routine seroconversion rate, demonstrate importance to improve intervention strategy to prevent feto-maternal CMV transmission.

## Background

Congenital cytomegalovirus (cCMV) infection is the most common congenital infection worldwide [1] representing the leading cause of sensorineural hearing loss (SNHL) in childhood and developmental delay [2–5]. Although most cCMV infections remain clinically undetected and infants are asymptomatic at birth, the risk is neurosensory sequelae which can lead to substantial developmental impairment in 10% of affected children [6]. The indirect effects of intrauterine infection, the ability of the placenta to provide oxygen and nutrients to the fetus has been impaired, causing the wide range of neurological symptoms [7]. Furthermore, a recent report implicates cCMV playing a role as an etiologic agent for childhood hematological malignancies [8].

Notably, awareness of the risk of CMV infection during pregnancy is rather low in pregnant women and even lower compared to awareness of rare diseases [3, 9, 10].

Particularly at risk of becoming infected with CMV is the CMV-seronegative pregnant woman (primary infection) who lives in the household with a young child up to three years of age. The virus is transmitted, for example, through infectious urine (diaper changes) or saliva (shared cutlery) of the infected child [11]. Unfortunately, basic prevention methods, such as vaccines, have not been shown to be effective [12–14]. Treatment options for CMV during pregnancy are limited and controversial (cytomegalovirus immunoglobulin or antiviral drugs) [15, 16]. The serial surveillance of the CMV serostatus with CMV Hyperimmunglobulin therapy was associated with a milde non-significant decrease in vertical CMV transmission rates in a European Phase III Randomized Trial [12].

Currently, CMV therapy during pregnancy with antiviral drugs is carried out as an Off-label-use (individual therapeutic trial) in Germany due to lack of validated treatment data [17, 18]. Prevention of maternal infection in CMV negative women is the best option to reduce the risk of fetal transmission [16]. Buxmann et al. conclude that counselling on hygiene measures may be the only effective method to prevent cCMV infections but worldwide awareness is low among women [19].

Currently, a general CMV IgG screening is not recommended for all pregnant women, neither in Germany nor in other European or international medical societies [17, 20, 21]. As a result, CMV screening during pregnancy is not covered by statutory health insurance in Germany. Instead, pregnant women can opt for a serological test as part of the individual health service (IGeL- Individuelle Gesundheitsleistung). These health services have to be paid for by patients themselves. Data are not available on the frequency of utilization of CMV serology testing during pregnancy as an individual health service.

The prevalence of CMV infection correlates with the rate of CMV seronegativity (absence of CMV IgG and IgM), socioeconomic status, geographic region and ethnicity [11, 22, 23].

As several studies demonstrated, the seronegativity rate in Germany was determined between 46% and 58% and decreases with age [24–26]. No validated prospective study data for CMV IgG seroconversion rate during pregnancy are available for Germany, but using international data derived from high seroprevalence population, the rate is estimated to be 0.5 [17, 27]. While a transmission rate to the unborn child of about 30–40% can be observed in primary CMV infections, this is markedly higher than in non-primary maternal infections (0.5-1%) [16, 19, 28].

After intrauterine virus transmission, either a clinically relevant CMV infection or subclinical infection can occur [17]. Approximately 10–15% o the newborns of primary infected women will be symptomatic at birth, e.g., they suffer from an intrauterine growth retardation (IUGR), microcephaly, or hepatosplenomegaly [29–31]. Most of the symptomatic newborns develop late complications. In particular a progressive SNHL, developmental delay, motor disabilities or vision impairment can occur [32, 33]. About 25% of all cases preenting congenital hearing loss and hearing disorders by the age of four years are caused by a CMV infection [34, 35].

However, women with persistent CMV IgG antibodies are still at risk for CMV reinfection or reactivation [36]. Most newborns of those secondary CMV infected women will be clinically unapparent [37]. Nevertheless, up to 15% of thse newborns also develop secondary diseases, most frequently manifested as SNHL [30, 31, 38]. Study data of high seroprevalence regions demonstrated the persistend risk of a secondary CMV infection during pregnancy. Published case reports of reinfected women have also demonstrated severe damage after CMV infections in their offspring [39–41].

The diagnosis of CMV primary infection is mainly based on the detection of IgG seroconversion, since CMV-IgM can be cross-reactive. To increase specificity, the diagnostic procedure should include analyzing the IgG avidity index to determine the time of infection [42–44].

The aim of the present retrospective observational study was (I) to obtain data on the CMV seroprevalence rate and (II) to analyze the rate of routine serological testing for CMV during pregnancy in a rural region of Germany.

## Methods

### Study population

For the retrospective observational study, data was collected from pregnant women between 1st of November 2014 and 30th of April 2018. The electronically stored data of these pregnant women originate from the medical laboratory “Prof. Schenk/Dr. Ansorge & Kollegen”, a main provider of laboratory tests in northern Saxony-Anhalt, a rural part of Germany. Between 2014 and 2018, an average of 8,728 live births per year were registered in this region [45, 46]. Thus, 30,548 live births were registered during the study period in Northern Saxony-Anhalt. Samples of 19,511 pregnant women in the outpatient sector were examined by the medical laboratory ‘Prof. Schenk / Dr. Ansorge & Kollegen` during the same period. This corresponds to about 64% of al live births in the study region that were available for analysis.

Inclusion criteria for study enrollment were at least one of the following pregnancy-mandatory laboratory tests during the study period: HIV antibody screening, Chlamydia trachomatis, or rubella IgG antibody screening. A total of 19,511 pregnant women were identified. In 18,460 (94.6%) of these women, health care was covered by statutory health insurance. In 1,051 (5.4%) cases, women were members of a private health insurance fund.

### CMV seronegativity rate and CMV serological testing as an individual health service

All cases with negative CMV IgG or IgM antibodies were defined as CMV seronegative. In Table 1 the cut-off reference ranges were shown for CMV IgG, IgM and IgG avidity (Table 1).

**Table 1:** Reference ranges to evaluate CMV IgG and IgM, IgG avidity

	Value (concentration)	Evaluation
CMV IgG^a^	< 12.0 U/ml	Negative
	12.0–14.0 U/ml	Borderline
	> 14.0 U/ml	Positive
CMV IgM^b^	< 18.0 U/ml	Negative
	18.0–22.0 U/ml	Borderline
	> 22.0 U/ml	Positive

CMV: Cytomegalovirus; IgG: Immunglobuline G, IgM: Immunglobuline M, U/ml: Units/Milliliter, Method: ^a^ Chemilumineszenz-Assay (CLIA), ^b^ Chemilumineszenz-Assay (CLIA),

^a^ Cytomegalovirus IgG Avidity (CLIA) Reference range: Low avidity: < 45.0%; grey zone: 45.0-54.9%; high avidity: ≥ 55.0%

In addition, the data were grouped by six billing types: hospital-based, statutory health insurance, private health insurance, individual health service, free of charge and unidentified. Unpaid cases are mostly based on a social indication. The individual health service (German Individuelle Gesundheitsleistungen, IgeL) is an additional diagnosis or treatment method that is not covered by the statutory health insurance. Insured persons must bear the costs of these tests at the doctor’s office themselves. The CMV IgG and IgM test for pregnant women in the outpatient setting is such an individual health service (out-of-pocket test) in Germany.

### CMV primary infection rate

To determine the rate of primary CMV infections during pregnancy, the cases with CMV IgG or IgM seroconversion and the results with positive or borderline CMV IgM were evaluated manually in the laboratory programme MIPS Vianova Labor (version 8.80, MIPS Deutschland GmbH & Co, KG; Walluf, Germany) of the medical laboratory “Prof. Schenk/Dr. Ansorge & Kollegen”. The following parameters were obtained: gestational age at the time of the serological testing, serological control tests, and avidity test of the IgG antibodies.

The CMV-infected cases were categorized into two groups. First, confirmed primary infection based on seroconversion or avidity testing of IgG antibodies in association with gestational age. For interpretation of the CMV IgG avidity testing cases were scored as follows: Low avidity was consistent with an infection acquired in the last 3 months. High avidity indicated an infection acquired > 3 months ago. Grey zone cannot be interpreted unambiguously. Secondly, potential primary CMV infections, including all cases where infection could not be ruled out for the following reasons: lack of a second serological test, no avidity test or no information on gestational age. The infection rate and the number of CMV tests were also evaluated depending on the billing types.

### Statistical analysis

The description of sample characteristics has the form mean ± standard deviation (SD) for continuous variables or n (%) for variables with discrete levels. Calculations were performed using IBM SPSS Statistics Version 26 (IBM Corporation, Armonk, New York, United States of America).

### Ethical approval

The institutional Ethics Committee of the Medical Faculty of the Otto-von-Guericke-University Magdeburg (17/16) and the local Ethics Committee Aerztekammer Sachsen-Anhalt (55/17), Germany, approved the study.

### Informed consent

Due to the retrospective nature of the study and the pseudonymisation of the data, the institutional and local ethics committee waived the requirement of informed consent.

## Results

### CMV seronegativity rate and CMV testing as an individual health service

3,800 and 2,470 of 19,511 women were tested for CMV IgG and IgM antibodies, respectively.

This corresponds to 19.5% of all pregnant women who had a laboratory test for CMV serological status. 1,635 women were tested for both CMV IgG and IgM at the same sample date. There are various indications for the simultaneous testing, for example hospitalized women with abnormal ultrasound findings or any signs of infection to the unborn child. 827 women were tested for CMV IgG and IgM at different sample dates. One reason for this could be that the statutory health insurance covers the costs for CMV IgM testing after a positive result in CMV IgG testing (which was covered by individual health service).

**Fig. 1:**
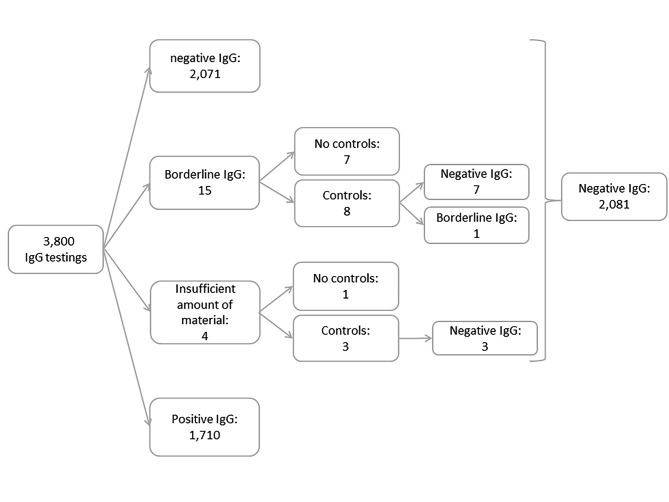
Overview of the results of the CMV IgG tests in the study population (IgG: Immunglobuline G; U/ml: Units/Milliliter, < 12.0 U/ml, Negative; 12.0–14.0 U/ml, Borderline; >14.0 U/ml, Positive)

The test results showed that 2,071 women had no CMV IgG antibodies and 1,710 women had positive IgG antibodies. Fifteen cases with a borderline IgG and four samples with less blood than necessary or missing control blood sample were registered. An overview of the study cases and the serological testing is shown in Fig. 1. After monitoring the serological control examinations, a total of 2,081 blood samples were negative for CMV IgG. Thus, the seronegativity rate of all pregnant women in the study period corresponded to 54.8%.

When classifying the 3,800 tests enrolled in the study by billing type, 2,710 cases were provided as an individual health service, 270 by a private health insurance provider, 669 by a statutory health insurance provider, 143 by a hospital, five free of charge and three with unidentified billing type.

During the study period, a total of 18,460 (94.6%) pregnant women were covered by statutory health insurance. 2,710 (14.7%) of these women opt for CMV testing as an individual health service. 270 (25.7%) of all women covered by private insurance providers requested the CMV IgG testing.

### CMV primary infection rate

**Fig. 2:**
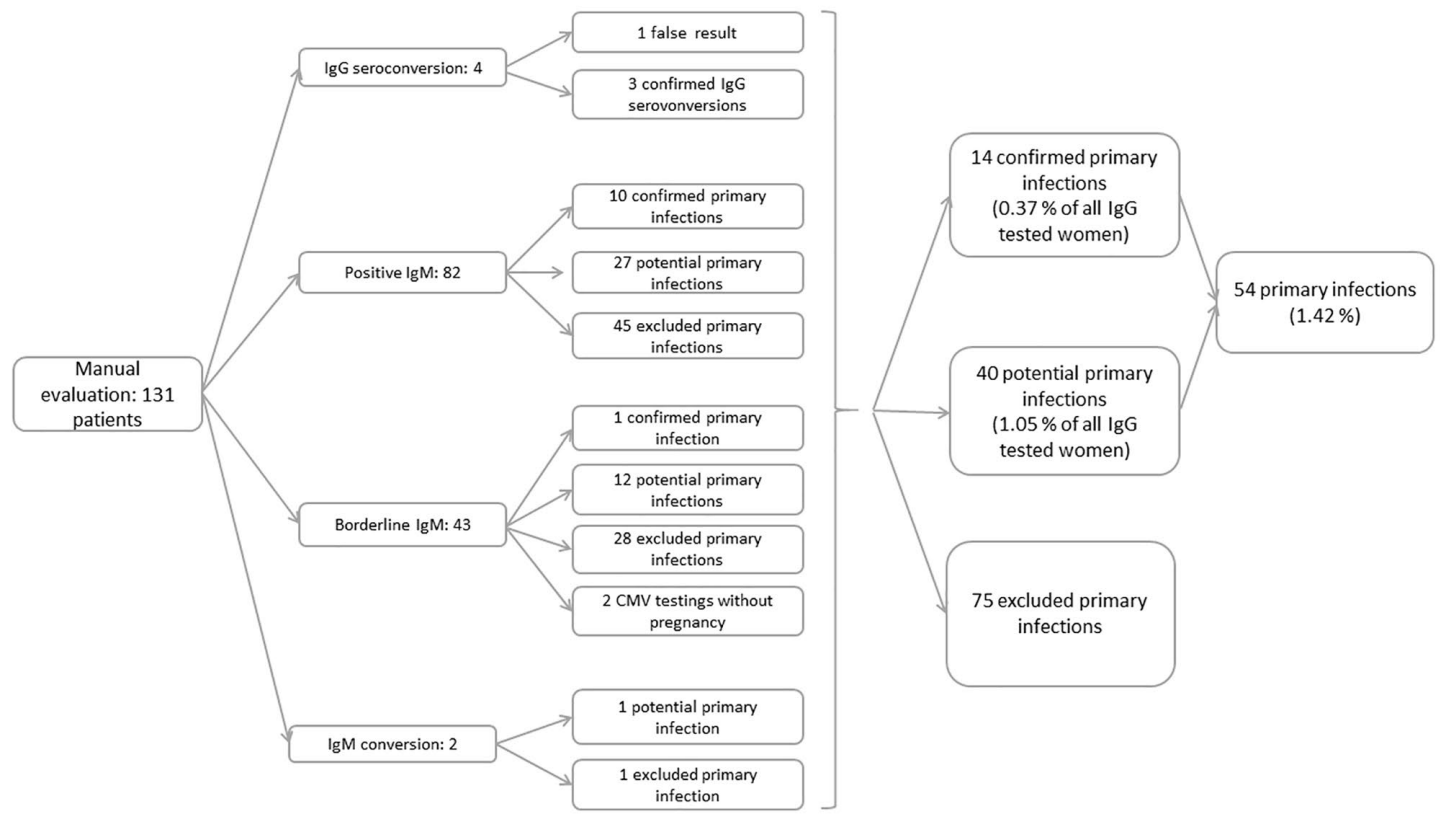
Flowchart of the evaluation process to distinguish between primary infection and borderline serological results in the study population following a step-by-step diagnostic assessment. (According to current German Diagnostic Guidelines [43] cases with CMV IgG or IgM seroconversion and the results with positive or borderline CMV IgM were evaluated manually. The following parameters were obtained: gestational age at the time of the serological testing, serological control tests, and IgG avidity test. Confirmed cases: Detection of CMV IgG seroconversion. CMV IgG seroconversion proves primary infection. Detection of low CMV IgG, low-avidity CMV IgG in combination with positive CMV IgM levels. Low CMV IgG avidity and positive IgM detection indicate a primary infection during the last three to four months.)

Overall, 131 cases with CMV IgG or IgM seroconversion, as well as cases with positive or borderline CMV IgM, were identified in the database (Fig. 2) [43].

The validation process of 131 electronically identified cases included a manual assessment of each case (Fig. 2). Based on this assessment, 14 confirmed and 40 potential CMV infection cases were detected, resulting in a CMV infection rate of 1.4% (n = 54 cases out of n = 3,800). In 75 cases, a CMV infection could be serologically excluded. The number of CMV serology tests and the results indicating an infection were stratified according to the billing method in Table 2.

**Table 2:** Overview on test results and CMV infection according to billing method

Billing type	IgG test n (%)	Number confirmed primary CMV infection n (%)	Number potential primary CMV infection n (%)	Total number of seroconversion n (%)
Statutory health insurance^a^	669 (100%)	8 (1.2%)	24 (3.6%)	32 (4.8%)
Hospitals	143 (100%)	1 (0.7%)	0	1 (0.7%)
Private health insurance	270 (100%)	0	6 (2.2%)	6 (2.2%)
Individual health service	2,710 (100%)	5 (0.2%)	10 (0.4%)	15 (0.6%)
Not known/ not applicable	8 (100%)	0	0	0
Total	3,800 (100%)	14 (0.4%)	40 (1.0%)	54 (1.4%)

CMV: Cytomegalovirus; IgG: Immunglobuline G

^a^ Statutory health insurance funds cover the IgG test in cases of suspected CMV infection to confirm the diagnosis

## Discussion

This retrospective study evaluated CMV seroprevalence rates in a sample of 19,511 pregnant women in rural Germany. The seronegativity rate of 54.8% determined in our study shows similar results to other published studies, which show a corresponding rate of 44-58% in women of childbearing age in Germany [24–26]. Hence, approximately half of all women of childbearing age from the covered population are at high risk of primary CMV infection during pregnancy. The calculated CMV seroconversion rate of 1.4% for our study population is similar to other countries with a comparable seroprevalence rate. Published data range from 1.2 to 4.1% [47].

Different studies demonstrated that hygiene measures could reduce the rate of maternal primary CMV infections [48]. Therefore, awareness of CMV infection during pregnancy represents an important parameter for the rate of seroconversion and, consequently, the number of CMV infections [49]. As shown by a study conducted in France, the rate of primary CMV infection during pregnancy is influenced by hygiene counseling [50]. Current research focuses on strategies for teaching pregnant or pregnant to be women and empowering them to adopt new habits to prevent CMV infections [51].

To the best of our knowledge, this paper is the first assessment of the routine uptake of CMV serological testing during pregnancy, classified by billing method. Our study demonstrates, that only 14.7% of pregnant women who are under the statutory health insurance scheme utilize this individual health service in outpatient settings. This indicates either lack of financial capacity or low awareness of the risk awareness of CMV infection, as has been shown in other studies [52, 53].

In the same period, serological tests for toxoplasmosis during pregnancy were requested approximately four times more frequently than CMV tests, 3,800 vs. 14,600 tests (internal evaluation of the medical laboratory, with reference to TW). However, the reasons for the reported discrepancy are not directly answered by the study data. One could presume a variable interaction of two clinical observations; namley toxoplasmosis may be more in the consciounce of women due to more vivid risk exposure (cats, raw food) and more propagation through the treating gynecologists [54, 55].

However, serological toxoplasmosis tests are only offered in the outpatient setting as part of an individual health service similar to CMV testing (at the woman’s own expense). Interestingly, the billing costs for serological toxoplasmosis tests are even higher than for CMV tests. Given these points, we assume that the low CMV testing rate is due to a lack of information on the risk of CMV infection during pregnancy than due to financial aspects.

Although, mortality due to cCMV is generally low [56]. The impact of cCMV as a leading non-genetic cause of SNHL [57, 58] and an important cause of neurodevelopmental delay in children worldwide has been demonstrated to be substantial [59–61]. This facts were barely appreciated when looking at published survey data over the past 10–15 years. In the 2005 survey of the United States population aged ≥ 18 years, women’s knowledge of the impact of CMV on the unborn child was lower than for any other disease or anomaly included in this published survey data [62]. In a recent report, our study group showed that the majority (60%) of pregnant women surveyed in a rural German region were still unaware of the risk of CMV infection in 2019 [63]. These findings are also compatible with data reported from other countries such as Canada, the Netherlands and Italy [55, 64, 65]. According to the published studies, pregnant women were more likely to be aware of other congenital infections, such as toxoplasmosis (93%), congenital anomalies such as trisomy 21 (95%), or fetal alcohol syndrome (55%) [52, 54, 66].

Based on the survey data in 2019, we observed a lower proportion of pregnant women accessed the serological CMV (25.0%) than the toxoplasmosis (72.3%) testing [63].

Cannon highlights in a review from 2009 the limited awareness of congenital CMV among clinicians and the women, most gynecologists do not counsel women about prevention of congenital CMV [67].

The serological CMV testing during pregnancy is not currently recommended as routine screening in European countries [68]. For example, the CMV testing is also not free of charge in Italy. Nevertheless, the study has shown that approximately 75% o all pregnant women requested the testing there. Furthermore, the proportion of screening tests increased significantly over time, from 60% to 2007 to 96% i 2014 [69]. This indicates the influence of health education and counseling of pregnant women, as has also been shown in other studies [70]. To conclude from various existing guidlines, counseling pregnant women is crucial, as hygiene education reduces seroconversion rates [17, 43, 68].

In our study, the seroconversion rate was 0.37–1.42% (Fig. 2); these results are in line with the rate of 2.0% described in a review by Hyde et al. [71].

Additionally, it was calculated that a maximum of 54 cases of CMV seroconversion during pregnancy could have been detected in our study period (with a seronegativity rate of 54.8%) if all 19,511 pregnant women in the included population had been tested for CMV infection. If we assume 10–15% of newborns are symptomatic, based on these seroconversions, we would expect up to 8 CMV-infected newborns in the covered study region. This would be in accordance with data previously reported from the same region [26]. Given the currently published evidence on treatment options to prevent maternal-fetal CMV transmission [72], it will be crucial to first detect all CMV seroconversions during pregnancy before finally reducing the burden of disease on the unborn child [73, 74]. The rate of transmission of intrauterine virus is higher in primary infections than in reinfection or reactivation of maternal CMV, estimated at between 30% and 40% [16, 36]. However, systematic surveillance data on trends in CMV testing during pregnancy and seroconversions rates are lacking in Germany [75].

The strength of our study was the ability to retrospectively monitor a large cohort of pregnant women from routine outpatient settings. Due to the retrospective study design, no.

demographic information or other known behavioral risk factors for CMV seroconversion and primary infection were asessed in the study population. The findings should be interpreted with caution, as the retrospective nature of our study did not allow for clinical follow-up and detection of secondary CMV infection in the pregnant women and their offspring. Nevertheless, our study results suggest that risk awareness of CMV infection during pregnancy may be an important factor that influences the performance of CMV testing. Several studies reported that pregnant women were more aware of other congenital infections such as toxoplasmosis and congenital anomalies such as trisomy 21 or fetal alcohol syndrome [54, 55]. In a survey conducted in the USA, only 6% of prgnant women reported that they were informed by their health care providers about the risk of CMV during pregnancy [9]. Likewise, other survey results have shown that an important source of information for pregnant and pregnant to be women is the consulting medical staff (doctors and midwives) [55, 76, 77].

To date, there are no evidence-based treatment options to prevent fetal CMV infection following CMV seroconversion during pregnancy [12]. Many questions remain unanswered. Reducing the CMV seroconversion rate through hygiene measures is the most promising prevention strategy proven in studies [68, 78]. Therefore, we currently see no other approach than improving risk awareness of CMV infection during pregnancy among women and medical staff. Future studies are needed to investigate awareness of fetal CMV infection and its socio-demographic determinants in pregnant women.

## Conclusions

Overall, the observed findings, seronegativity rates in conjunction with seroconversion rate obtained from routine outpatient settings emphasize the importance of developing an intervention strategy to prevent the feto-maternal transmission of CMV infection.

## Data Availability

The data that support the findings of this study are not openly available due to the sensitive nature of the questions asked in this study and are available from the corresponding author upon reasonable request.
